# Expression of Ror2 Mediates Invasive Phenotypes in Renal Cell Carcinoma

**DOI:** 10.1371/journal.pone.0116101

**Published:** 2014-12-26

**Authors:** Neal R. Rasmussen, Zufan Debebe, Tricia M. Wright, Samira A. Brooks, Adam B. Sendor, A . Rose Brannon, A . Ari Hakimi, James J. Hsieh, Toni K. Choueiri, Pheroze Tamboli, Jodi K. Maranchie, Peter Hinds, Eric M. Wallen, Catherine Simpson, Jacqueline L. Norris, William P. Janzen, W. Kimryn Rathmell

**Affiliations:** 1 Curriculum in Genetics and Molecular Biology, University of North Carolina at Chapel Hill, Chapel Hill, North Carolina, 27599, United States of America; 2 Lineberger Comprehensive Cancer Center, University of North Carolina at Chapel Hill, Chapel Hill, North Carolina, 27599, United States of America; 3 Curriculum in Toxicology, University of North Carolina at Chapel Hill, Chapel Hill, North Carolina, 27599, United States of America; 4 Human Oncology and Pathogenesis Program, Memorial Sloan Kettering Cancer Center, New York, New York, 10065, United States of America; 5 Department of Surgery, Urology Service, Memorial Sloan Kettering Cancer Center, New York, New York, 10065, United States of America; 6 Department of Medical Oncology and Kidney Cancer Center, Dana Farber Cancer Institute, Boston, Massachusetts, 02215, United States of America; 7 Department of Pathology, MD Anderson Cancer Center, Houston, Texas, 77030, United States of America; 8 Department of Urologic Oncology, University of Pittsburgh Medical Center, Pittsburgh, Pennsylvania, 15219, United States of America; 9 Department of Urology, University of North Carolina at Chapel Hill, Chapel Hill, North Carolina, 27599, United States of America; 10 Department of Epidemiology, University of North Carolina at Chapel Hill, Chapel Hill, North Carolina, 27599, United States of America; 11 Center for Integrative Chemical Biology and Drug Discovery, University of North Carolina at Chapel Hill, Chapel Hill, North Carolina, 27599, United States of America; 12 Departments of Medicine and Genetics, University of North Carolina at Chapel Hill, Chapel Hill, North Carolina, 27599, United States of America; Southern Illinois University School of Medicine, United States of America

## Abstract

Ror2 is a Wnt ligand receptor that is overexpressed in a variety of tumors including clear cell renal cell carcinoma (ccRCC). Here we demonstrate that expression of wild type Ror2 results in increased tumorigenic properties in *in vitro* cell culture and *in vivo* xenograft models. In addition, Ror2 expression produced positive changes in both cell migration and invasion, which were dependent on matrix metalloprotease 2 (MMP2) activity. Mutations in key regions of the kinase domain of Ror2 resulted in the abrogation of increased tumor growth, cell migration, and cell invasion observed with expression of wild-type Ror2. Finally, we examined Ror2 expression as a prognostic biomarker for ccRCC utilizing the TCGA ccRCC dataset. High expression of Ror2 showed a significant correlation with higher clinical stage, nuclear grade, and tumor stage. Furthermore, high expression of Ror2 in ccRCC patients correlated with significant lower overall survival, cancer specific survival, and recurrence free survival. Together, these findings suggest that Ror2 plays a central role in influencing the ccRCC phenotype, and can be considered as a negative prognostic biomarker and potential therapeutic target in this cancer.

## Introduction

Renal cell carcinoma (RCC) remains a growing problem worldwide, as its incidence and mortality rate continue to climb steadily at ∼2–3% per decade [1]. In the United States in 2013, it is estimated there will be over 65,000 new cases and 13,000 deaths, with nearly one-third of these patients presenting with metastatic RCC [2]. For those patients with metastases upon diagnosis, the 5-year survival rate remains only 5–10% [1], [3]. RCC consists of several subtypes, the most prevalent being clear cell renal cell carcinoma (ccRCC), which accounts for ∼70% of cases. ccRCC is notoriously difficult to treat as it is relatively radioinsensitive and highly unresponsive to traditional chemotherapeutic approaches. The advent of targeted therapeutics have improved the outlook for ccRCC patients, yet their efficacy remains limited mainly to improvements in progression free survival as opposed to overall survival. As such, there is an urgent necessity to identify novel therapeutic targets that contribute to tumor progression and have the potential to serve as prognostic biomarkers in ccRCC.

An exciting therapeutic target recently identified in ccRCC is the developmentally regulated, receptor tyrosine kinase-like orphan receptor 2 (Ror2) [4]. Although, early work showed Ror2 expression to be largely restricted to early embryogenesis with its mutation or loss resulting in various skeletal malformations in humans and mice [5], [6], [7], its expression has been reported in an increasing array of cancers including osteosarcoma, melanoma, prostate cancer, gastric cancer, gastrointestinal stromal tumor (GIST), leiomyosarcoma (LMS), colorectal cancer, squamous cell carcinoma of the head and neck and ccRCC [4], [8], [9], [10], [11], [12], [13], [14], [15], [16]. We have observed that Ror2 can participate in canonical beta-catenin growth promoting signals in cell lines, indicating that the cells are poised for pathway activation in response to Wnt ligand engagement [17]. However, aberrant expression of Ror2 has been shown to promote migration, invasion, and metastasis, in addition to cell proliferation, mirroring some of its roles in early development [4], [8], [9], [11], [13], [18], [19]. Some of these Ror2 dependent effects of increased cell motility and invasive capability have been suggested to be mediated through its regulation of matrix metalloprotease (MMP) expression which are enzymes responsible for degradation of the surrounding extracellular matrix (ECM) [8], [20]. The regulation of various members of the MMP family by Ror2 has been shown to be highly dependent upon the cell context. The differing effects of these various contextual spheres is well illustrated in osteosarcoma cells where Ror2-dependent expression of MMP13 has been shown to be mediated through either through Dvl2 and Rac1 in SaOS-2 cells or Dvl3 in U2-OS cells [18]. Further, observations of the aberrant expression of Ror2 in prostate cancer and RCC cells have shown alterations in MMP1 and MMP2 expression, respectively [4], [13].

In addition to its tumor promoting role, prior studies have suggested Ror2's potential as a prognostic biomarker, with high Ror2 expression correlating with surgical stage and tumor metastasis in osteosarcoma [21], metastatic melanoma [19], [22], and poorer clinical outcome in colorectal cancer, GIST and leiomyosarcoma [11], [16]. Because earlier work has shown that Ror2 expression is associated with tumor growth phenotypes in ccRCC cells, we sought to expand our understanding of the tumor promoting role of Ror2 in ccRCC [23]. To do this, we explored cell phenotypes related to MMP2 expression and activity, as well as tumor cell invasive capacity. We also explored the effects of Ror2 overexpression in tumor xenograft growth, and in The Cancer Genome Atlas (TCGA) ccRCC tumor datasets to determine how Ror2 expression related to clinical outcomes.

## Results

### Expression of Ror2 promotes in vivo tumor growth and invasion

**Figure 1:**
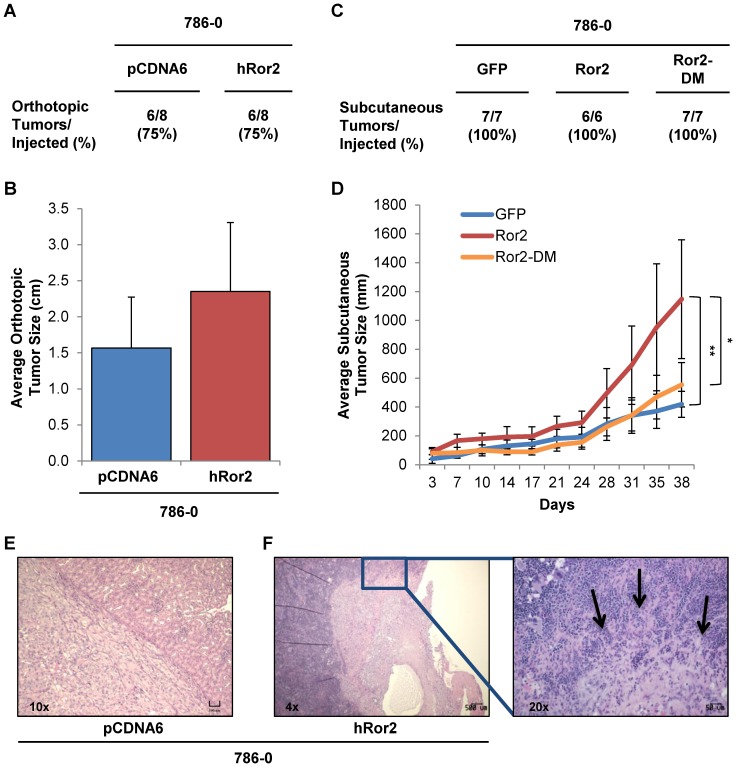
Expression of Ror2 promotes *in vivo* tumor growth and invasion. **A&C**) Tabulated rates of successful injection and growth of all 786-0 xenograft tumors are shown. **B**) Average tumor size (cm) with error bars showing stdev for resulting xenograft tumors following paired orthotropic injections of 786-0 cells containing either the control empty vector, pcDNA6 or hRor2 exhibited a trend to increased *in vivo* tumor growth with Ror2 overexpression. **D**) Average tumor size (mm) with error bars showing stdev for resulting xenograft tumors following subcutaneous injection of 786-0 cells containing either the control empty vector, GFP, wild-type Ror2, or Ror2-DM showed a significant increase in *in vivo* tumor growth with wild-type Ror2 overexpression. *P*-values were calculated using an Anova one-way analysis (*<.05 **<.01). **E**) Representative image of an orthotopic xenograft consisting of 786-0 vector control cells. **F**) Increased invasion of local tissues was evident with Ror2 expression, with arrows highlighting areas of continued invasion into the spleen.

The aberrant expression of Ror2 has been shown to serve in a tumor promoting role in variety of cancers, including RCC. Prior work in RCC has shown that suppression of Ror2 with shRNA in RCC cells orthotopically injected into the kidney resulted in a dramatic reduction of tumor growth *in vivo* [4]. However the effects of overexpression of Ror2 in RCC cells *in vivo* have yet to be elucidated. To examine the effects of Ror2 overexpression, we utilized 786-0 cells expressing either empty vector controls or Ror2 in both orthotopic and subcutaneous xenograft models. We selected an established cancer cell line, which expresses low levels of Ror2 basally and is known to display a xenograft growth phenotype, anticipating that Ror2 expression might modify the tumorigenic phenotype rather than act overtly as an oncogene. Moreover, our exploration of cell lines has demonstrated a wide range of Ror2 expression in cancer cell lines, with 786-0 RCC cells at the low end of the spectrum (S1 Fig.). Expression of Ror2 in 786-0 cells had no effect on the rate of tumor formation in comparison to the controls (Fig. 1A and C). However, Ror2 overexpressing 786-0 cells injected orthotopically into the kidney showed a trend towards increased tumor size (Fig. 1B).

To further examine this trend, we generated subcutaneous xenografts using 786-0 cells expressing either a GFP control, wild-type Ror2 (Ror2), or mutated Ror2 (Ror2-DM), which was modified in two key regions known to be critical for the putative kinase domain: the ATP binding pocket and the DFG (found as DLG in Ror2) loop [17]. Consistent with our findings with the orthotopic model, we observed a significant increase in tumor size with expression of wild-type Ror2 over the control. However, the expression of Ror2-DM in 786-0 cells abrogated the increased tumor growth seen with wild-type Ror2 (Fig. 1D), suggesting that this domain retains a component of the activity for Ror2 to promote tumor growth.

In addition to Ror2's role in promoting tumor growth, it has been shown to play a role in cell migration and invasion. To assess Ror2 invasive potential, H&E sections for each orthotopic xenograft were examined. Although, invasion of the kidney capsule was observed in both control and Ror2 expressing tumors, only Ror2 overexpressing cells exhibited invasion into surrounding tissue as seen in Fig. 1F (an example of tumor invasion into the spleen).

### Migration in RCC cells is enhanced by Ror2 expression

**Figure 2:**
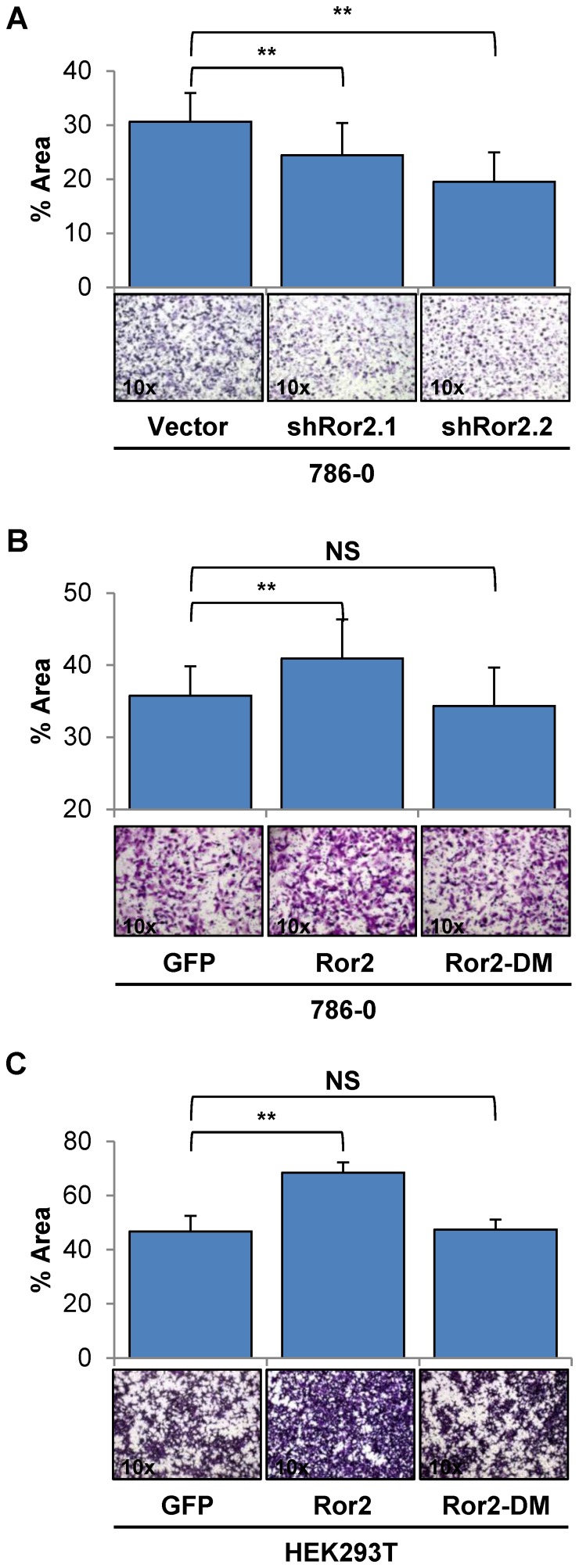
Migration in RCC cells is enhanced by Ror2 expression. Analysis of migration potential using Boyden chamber assays in **A**) 786-0 cells show a significant reduction in migration in both independent shRNA Ror2 knockdowns in comparison with 786-0 vector control cells, with representative images shown below. Boyden chamber assays using **B**) 786-0 & **C**) HEK293T cells under normal culture conditions show a significant reduction in migration with mutant Ror2-DM cells compared to Ror2 expressing cells, with representative images shown below. The percentage area covered was quantitated from random fields using ImageJ. *P*-values were calculated using an Anova one-way analysis with error bars representing stdev (*<.05, **<.001).

Following our observations of Ror2 leading to increased tumor growth and invasion *in vivo,* we sought to more fully elucidate the role of Ror2 mediating cell migration and invasion in RCC. To clarify the contribution of Ror2 to cell migration in RCC, we utilized a Boyden chamber assay, allowing us to observe single cell motility as cells migrated across the membrane in response to extracellular signaling cues. Both 786-0 shRor2 knockdown cell lines exhibited reduced rates of cell migration in comparison to 786-0 vector control cells, in agreement with previous studies using a scratch wound healing assay [4] (Fig. 2A).

To further determine if Ror2's contribution to the migratory phenotype in RCC cells was dependent upon the kinase domain, we used 786-0 cells which overexpressed either wild-type Ror2 or mutant Ror2-DM. We observed the migratory potential of these cells in a Boyden chamber assay under normal culture conditions and found that 786-0 cells expressing Ror2-DM exhibited a migratory rate similar to GFP control cells, whereas the 786-0 cells expressing wild-type Ror2 again exhibited a significant increase in cells that migrated across the membrane (Fig. 2B). We also examined the effects of expression of wild-type or mutant Ror2 in HEK293T cells, a transformed embryonic kidney cell line which does not basally express Ror2 (S1 Fig.), where we observed a mirrored increase in migration only in cells expressing wild-type Ror2 relative to GFP control (Fig. 2C).

### Expression of Ror2 mediates cell invasion in RCC cells

**Figure 3:**
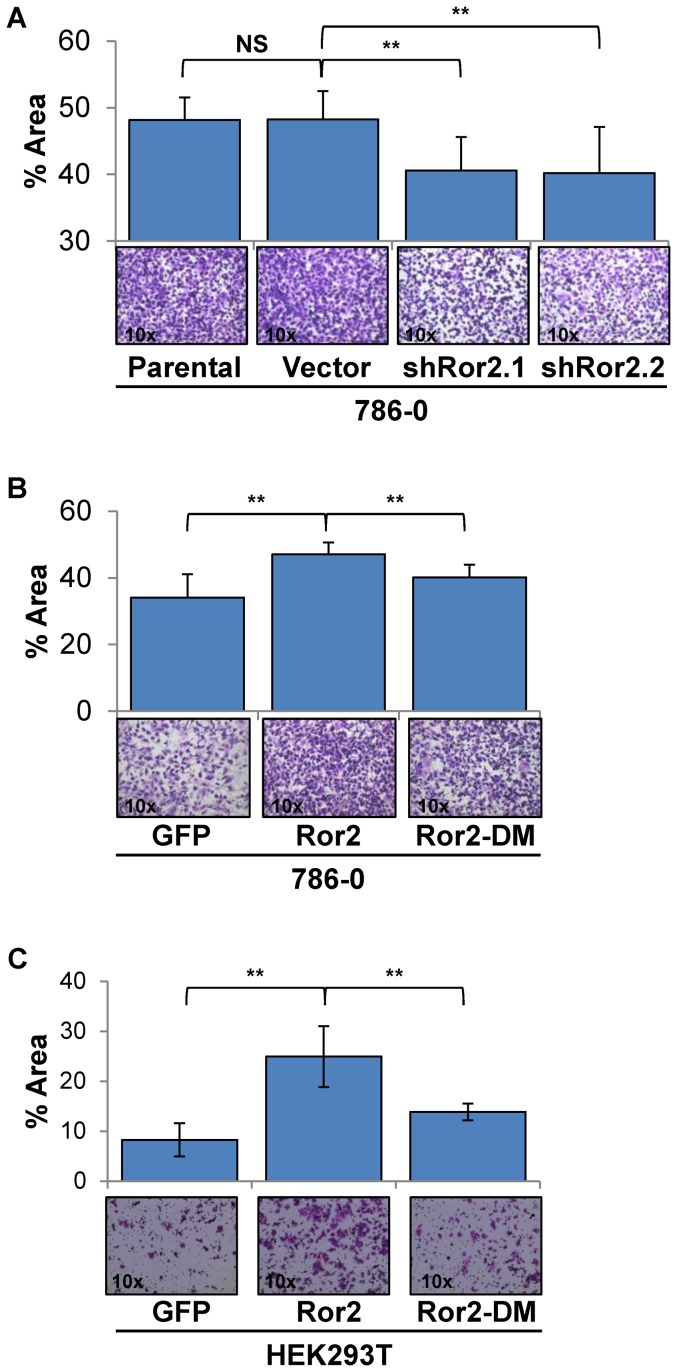
Ror2 expression regulates cell invasion in RCC cells. Analysis of invasive potential using Boyden matrigel coated chamber assays under normal culture conditions with **A**) 786-0 cells show a significant reduction in invasion in both independent shRNA Ror2 knockdowns in comparison with parental 786-0 and vector control cells (representative images shown below). Additionally, **B**) 786-0 & **C**) HEK293T cells under normal culture conditions show a significant increase in cell invasion with overexpression of wild-type Ror2 that is reduced with expression of Ror2-DM (representative images shown below). The percentage of area covered was quantitated from random fields using ImageJ. *P*-values were calculated using an Anova one-way analysis with error bars representing stdev shown (*<.05, **<.001).

For metastasis to occur, both migratory capacity and the ability to degrade the extracellular matrix are necessary. Expression of Ror2 has been shown to mediate expression of several MMPs, which may contribute to cell migratory/invasive phenotypes in a multitude of cancers including RCC [4], [8], [13], [18], [20]. Building on our findings of Ror2 expression leading to increased invasion in xenografts, we sought to examine the effects of Ror2 expression on cell invasion under controlled conditions *in vitro* using the Boyden chamber assays, this time coated with matrigel. Similar to our cell migration experiments, we observed a marked reduction of invasion in both shRor2 knockdown cell lines in comparison to parental 786-0 and 786-0 vector control cells (Fig. 3A). Furthermore, overexpression of wild-type Ror2, resulted in a significant increase of cell invasion in 786-0 and HEK293T cells that was reduced in cells expressing mutant Ror2-DM (Fig. 3B and C). These results demonstrate that Ror2 expression plays an integral part in promoting cell motility and invasion in RCC cells.

### Ror2 dependent expression of MMP2 mediates cell invasion in RCC cells

**Figure 4:**
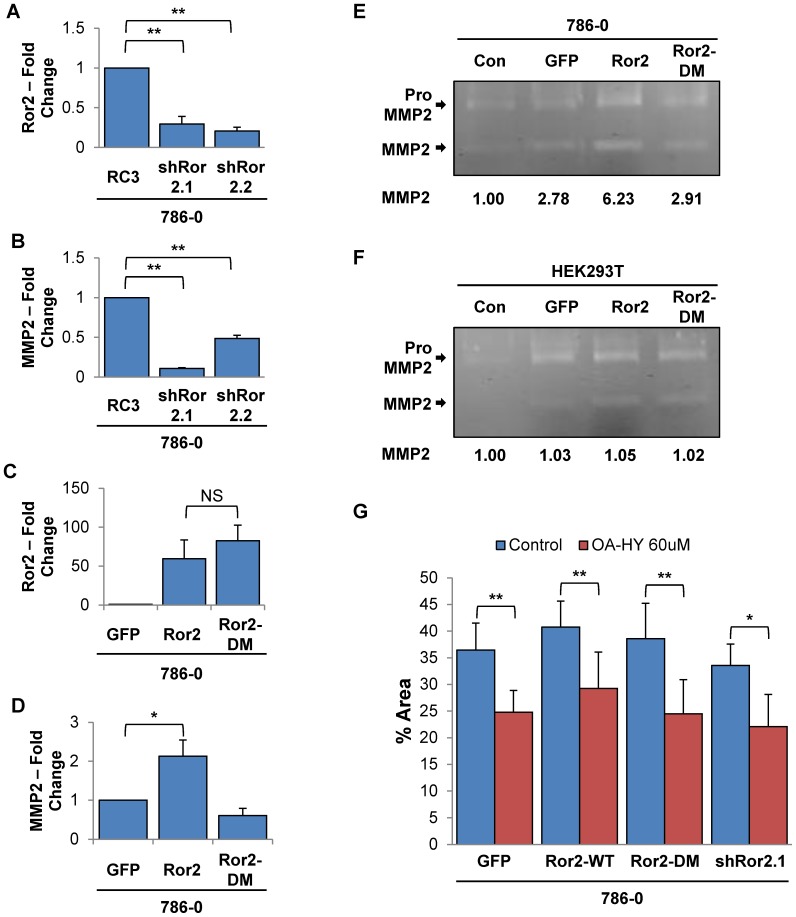
Ror2 regulation of MMP2 expression in RCC cells is dependent upon an intact kinase domain. Quantitative RT-PCR for 786-0 cells shows a significant correlated decrease in **A**) Ror2 and **B**) MMP2 mRNA levels in both shRNA knockdown cell lines relative to control. **C**) Additionally, induction of wildtype Ror2 and Ror2-DM is evident following treatment with doxycycline (500 ng/ml). **D**) But a matching increase in MMP2 is only seen with overexpression of wildtype Ror2. For all quantitative RT-PCR assays, transcript values were normalized to β-actin RNA internal standard with fold change calculated in reference to either 786-0 control or unstimulated GFP expressing cells. Error bars represent SEM across triplicates of a representative duplicated experiment. Gelatin Zymography shows increased levels of both the precursor pro-MMP2 and its cleaved active form with overexpression of wildtype Ror2 relative to GFP vector control in both **E**) 786-0 and **F**) HEK293T cells. Quantification of the levels of active MMP2 normalized to the media only control (Con) is shown below each gel. **G**) Invasion of 786-0 cells seeded in Boyden matrigel coated chambers under normal culture conditions show a significant decrease upon treatment with MMP2 inhibitor (OA-Hy - 60 uM) in both independent shRNA knockdowns and Ror2 overexpression in comparison with vehicle control. The percentage of area covered was quantitated from random fields using ImageJ. Error bars represent stdev across duplicates of a representative duplicated experiment. *P*-values were calculated using an Anova one-way analysis (*<.05, **<.001).

Prior work in our lab has shown the expression of matrix metalloprotease 2 (MMP2), an extracellular matrix (ECM) remodeling protease, is regulated at the transcriptional level by Ror2 in RCC cells [4]. In addition, Ror2*^+/−^* mice exhibited decreased MMP2 levels in damaged kidneys undergoing epithelial-to-mesenchymal transition (EMT) following unilateral ureteral obstruction-induced fibrosis [24]. Therefore, we hypothesized that Ror2's effects on cell invasion were likely mediated through MMP2. We first examined the Ror2 dependent expression of MMP2 in 786-0 cells utilizing quantitative RT-PCR, which confirmed that suppression of Ror2 in both of our knockdown lines resulted in a marked reduction of MMP2 expression (Fig. 4A and B). We also observed that overexpression of wildtype Ror2 resulted in increased expression of MMP2, but expression of Ror2-DM failed to increase basal expression of MMP2 (Fig. 4C and D).

We utilized gelatin zymography to explore the availability of active soluble MMP2 protein in these cells, as it is cleaved post-translation for activity. However, both the pro and active form of MMP2 are detected by this procedure as the developing reagents allow both forms to actively degrade the gelatin within the SDS gel, resulting in two clear bands following Coomassie staining. In agreement with our quantitative RT-PCR findings, quantification of the cleaved active form of MMP2 in 786-0 and HEK293T cells overexpressing wild-type Ror2 showed increased MMP2 levels. However, Ror2-DM induced MMP2 levels equivalent to GFP control cells (Fig. 4E and F). To further determine whether Ror2 effects on cell invasion were mediated through MMP2, RCC cells seeded in matrigel-coated Boyden chambers were treated with 60 uM *cis-*9-Octadecenoyl-N-hydroxylamide (an MMP2 inhibitor) or vehicle. Treatment with the MMP2 inhibitor resulted in reduced migration in each of the RCC cell lines (Fig. 4G), demonstrating that MMP2 activity does contribute to the Ror2 invasive phenotype.

### High Ror2 expression correlates with increased tumor growth and stage in primary human ccRCC tumors

To determine if our findings with Ror2 promoting aggressive features of tumor growth, cell migration, and cell invasion translated into promoting tumor progression in ccRCC patients, we turned to the recently published TCGA ccRCC dataset [25]. The scope and size of the TCGA ccRCC dataset of over 400 tumors with complete clinical and molecular information provided us with significant statistical power to explore Ror2's contribution to the underlying molecular biology and provide a more complete picture of ccRCC tumorigenesis. Using the ccRCC TCGA dataset, we classified tumors into Ror2-High and Ror2-Low expression categories (see methods). The clinical and pathological characteristics of these patients are summarized in Table 1.

**Table 1:** Summary of clinical characteristics of patients in the TCGA ccRCC dataset.

	TCGA	Ror2-High	Ror2-Low	P-value
Sample (n)	468	234	234	
**Age at Diagnosis**	61 (26–90)	60 (26–90)	61 (29–86)	0.0933
**Gender**				**1.12×10^−5^**
** Male**	306 (65%)	176 (75%)	130 (55%)	
** Female**	162 (35%)	58 (25%)	104 (44%)	
**Prior Tumor (Yes)**	126 (27%)	69 (29%)	57 (24%)	0.2516
**Clinical Stage**				
** Stage I**	224 (48%)	101 (43%)	123 (53%)	-
** Stage II**	45 (10%)	22 (9%)	23 (10%)	0.7433
** Stage III-IV**	199 (43%)	111 (47%)	88 (38%)	**0.0322**
**Grade**				
** G1–G2**	205 (44%)	84 (36%)	121 (52%)	-
** G3**	185 (40%)	93 (40%)	92 (39%)	0.0678
** G4**	72 (15%)	57 (24%)	15 (6.4%)	**2.18×10^−8^**
** GX**	6 (1.3%)	0 (0%)	6 (2.6%)	-
**Tumor Size (cm)**	6.57	6.90	6.24	0.023
**Staging (TNM)**				
** T1**	228 (49%)	103 (44%)	125 (53%)	-
** T2**	58 (12%)	25 (11%)	33 (14%)	0.8826
** T3–T4**	182 (39%)	106 (45%)	76 (32%)	**0.0098**
**Nodes**				
** Node − (N0)**	226 (48%)	110 (47%)	116 (50%)	-
** Node + (N1)**	16 (3.4%)	9 (3.7%)	7 (2.9%)	0.5598
** Node unknown (NX)**	226 (48%)	115 (47%)	111 (46%)	-
**Metastasis**				1.0
** Mets − (M0)**	388 (%)	191 (82%)	191 (82%)	
** Mets + (M1)**	69 (%)	43 (18%)	43 (18%)	

**Figure 5:**
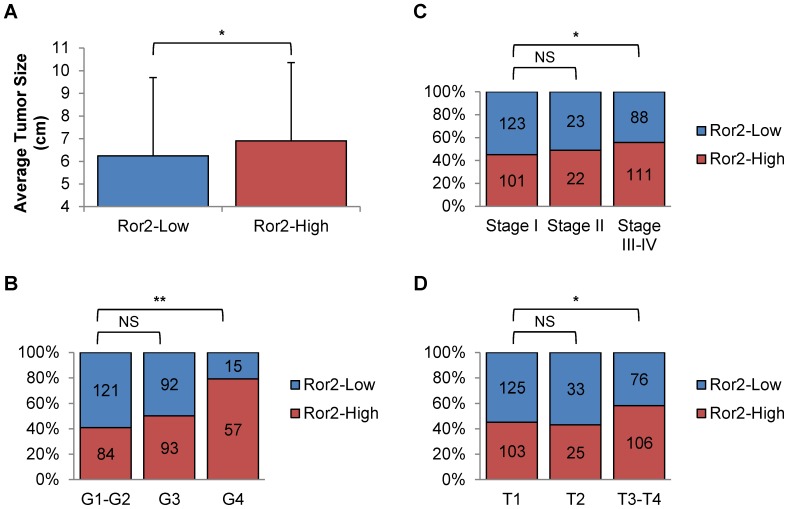
High Ror2 expression correlates with increased tumor growth and stage in primary human RCC tumors. Tumor percentage breakdowns of TCGA ccRCC cases (468) stratified by Ror2-High and Ror2-Low expression show Ror2-High tumors exhibiting significant increases in **A**) tumor size, **B**) higher nuclear grade, **C**) clinical stage, and **D**) tumor stage. *P*-values were calculated using either an Anova one-way analysis or Fisher's exact test (*<.05, **<.001).

Reflecting our previous results showing Ror2 expression effecting tumor growth in xenografts, we examined the TCGA dataset and found a corresponding increase in mean tumor size (cm of largest diameter) in Ror2-High expressing patients, which was significant in spite of the large standard deviations around tumor size (*P* = 0.023) (Fig. 5A). We also observed a greater fraction of Ror2-High expressing tumors within the highest nuclear grade tumors (G4) (*P* = 2.18×10^−8^) in comparison to low nuclear grade (G1–G2) (Fig. 5B). To determine if Ror2-High expressing tumors correlated with increased disease severity as seen with osteosarcoma [21], we analyzed to the TCGA dataset and found a significant increase in Ror2-High expressing tumors in both advanced tumor stage (T4) (*P* = 0.0098), which is associated with local invasion and late clinical stage (Stage III–IV) (*P* = 0.0322) (Fig. 5C & D).

### Ror2 expression predicts poor clinical outcome in patients with ccRCC

Because higher Ror2 expression has a prior mentioned potential to serve as an independent biomarker in osteosarcoma, colorectal cancer, GIST,leiomyosarcoma, and as part of 34 gene panel in ccRCC we sought to determine whether Ror2 expression could serve as an independent prognostic factor in ccRCC [11], [16], [21], [26]. This information would not only be a valuable contribution to prognostic nomograms, but has the potential to develop as a predictive biomarker upon advent of Ror2-directed therapies in ccRCC.

**Figure 6:**
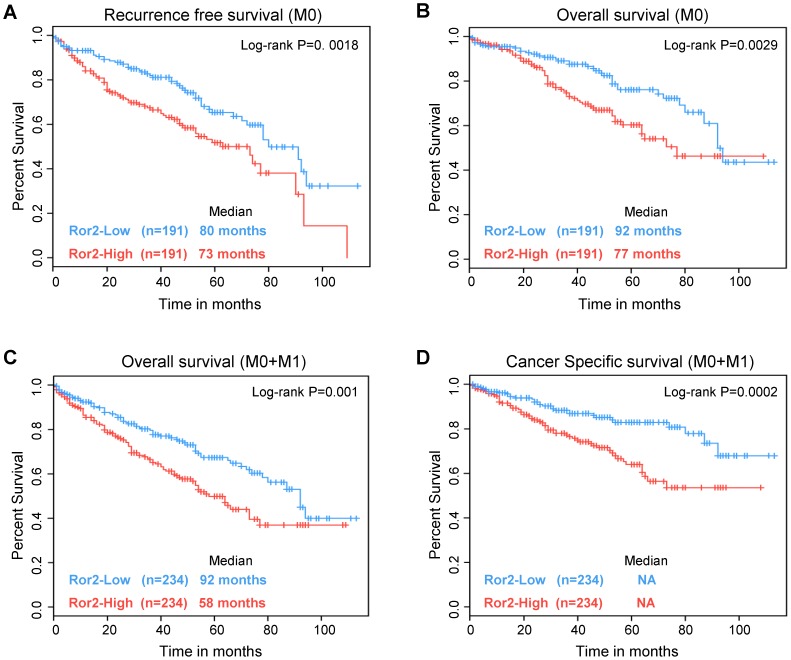
Ror2 expression predicts poor clinical outcome in patients with RCC. Kaplan-Meier survival curves for ccRCC cases from the TCGA were stratified by Ror2 expression. Ror2-High expressing ccRCC tumors exhibited a significant lower **A**) recurrence-free survival and **B**) overall survival in non-metastatic patients (M0) (N = 382). Likewise, an analysis with all RCC bearing patients (M0+M1) (N = 468) showed a significant decrease in **C**) overall survival and **D**) cancer specific survival with Ror2-High compared Ror2-Low. Log-rank p-values were assigned using a univariate cox regression analysis.

We first analyzed overall survival for either Ror2-High or Ror2-Low expression from the ccRCC TCGA tumors with 95% confidence intervals and the median survival. Using a univariate Cox regression analysis, we examined recurrence-free survival in all non-metastatic patients (M0) (n = 382) and found that the Ror2-Low subtype had a significant survival advantage over Ror2-High patients (*P* = 0. 0018) (Fig. 6A). Likewise, we also found that Ror2 expression predicated a significant difference in overall survival in both non-metastatic (M0) (n = 382) (*P* = 0.0029) and all patients (M0+M1) (n = 468) (*P* = 0.001) (Fig. 6B and C). In addition, an analysis of cancer specific survival in all ccRCC patients found that patients within the Ror2-Low subtype had significantly longer survival times (*P* = 0.0002) (Fig. 6D). These results show Ror2 expression is correlated with more aggressive and invasive disease, and that Ror2 can contribute to developing prognostic models as well as performing as an independent biomarker.

## Discussion

As RCC is generally asymptomatic in early stages, over a third of patients are diagnosed with metastatic or advanced disease. In addition, RCC tumors can grow to an enormous size without developing invasive features, such that molecular features must be present to alter their tumor progression from continued non-invasive growth to tumors with the potential for either local or distant invasion and metastasis. An expanding knowledge of the underlying molecular biology of ccRCC, which is dominated by mutations and/or loss of pVHL leading to constitutive stabilization of the HIF transcription factors and expression of a host of target genes, has led to the successful development of several lines of targeted therapeutics. However, there remains considerable room for improvement as most patients still succumb to the disease. We sought to further elucidate the role of Ror2 as an additional tumor promoting component in ccRCC, focusing on its effects in enhancing tumor cell invasive features.

Prior work had shown that knockdown of Ror2 in RCC cells resulted in significant loss of tumor growth in xenografts [4]. However, this analysis left unanswered, if further alterations, in Ror2 expression or sequence would also result in changes in tumor growth or impact the invasive features of these tumors. We show that overexpression of Ror2 in RCC cells resulted in increases in tumor growth as determined by average daily tumor size, as well as the observation of increased local invasion of xenograft tumors. In addition, disruption of the kinase domain of Ror2 through site directed mutagenesis resulted in the abrogation of this increased tumor growth. These findings not only show Ror2 promotes tumor growth, but that the kinase domain is needed to support this activity. Yet, whether the suppressive impact we have observed here on tumor growth, cell migration and invasion following modifications within the kinase domain are due to changes affecting its catalytic activity are unknown. Recent work has established the paralog Ror1 as a pseudokinase with no inherent catalytic activity, but with its suppression still resulting in decreased cell proliferation, anchorage independent growth, and tumor growth [27]. With Ror1 and Ror2 showing conservation in key residue changes in the kinase domain, Ror2's potential status as pseudokinase remains an open question in the field [28]. Ror2 may be serving purely as a scaffolding protein or allosteric regulator, whereby these mutations may be interfering with its effective binding and regulation by other intracellular signaling partners mediating these tumor phenotypes. It is also possible that Ror2 may have weak inherent catalytic activity that would potentially be disrupted by these kinase domain mutations leading to alterations in downstream signaling. Our results cannot exclude either of these possibilities and, as such, further studies are needed to more fully elucidate Ror2's role as a kinase and its signaling cascades in promoting tumor growth and invasion in RCC.

While our previous studies have shown that suppression of Ror2 in RCC cells did not alter cell proliferation *in vitro*, the increases in tumor size observed in wild-type Ror2 overexpressing xenografts may be the result of several possibilities [4]. First, it is possible the increased cell motility and ability to invade beyond the local tissue allows introduction to new tumor microenvironment and exposure to additional proliferative signals. It is also possible that although Ror2 expression did not alter cell proliferation under *in vitro* culture conditions, that Ror2 expressing cells *in vivo* may be presented with additional signaling cues leading to signaling cascades enhancing tumor growth.

In addition to Ror2 promoting tumor growth, we also observed changes consistent with Ror2 dosage correlated effects on both cell migration and invasion. Prior work in RCC showed the ECM remodeling protein MMP2 expression to be Ror2 dependent in RCC cells and correlated with Ror2 expression in human ccRCC tumors [4]. Our results using quantitative RT-PCR and gelatin zymography were in agreement with these past findings. Although the consistent increased expression of MMPs has been noted with Ror2 expression now in several cell contexts, here we have uniquely established the need for the Ror2 kinase domain in maintaining heightened MMP2 expression and activity. Furthermore, we have shown the increases in cell invasion seen with Ror2 overexpression are lost with targeted inhibition of MMP2 using a pharmacologic inhibitor. These results suggest that Ror2 mediates, at least a portion of, these cells ability to migrate invasively via MMP2 activity. However as inhibition of MMP2 resulted in decreased invasion in all paneled cell lines including Ror2 knockdown demonstrates that varying levels of active of MMP2 could be found in each cell line. Although changes in Ror2 expression resulted in an augmentation of MMP2 expression, these results suggest that Ror2 probably works in conjunction with a host of competing factors including HIF which are known to regulate its expression. These results, combined with previous findings linking Ror2 to tumor growth, migration, and cell invasion phenotypes are suggestive of Ror2's role in promoting tumor progression and enhancing invasive growth in ccRCC.

These changes in both tumor growth and cell invasion seen experimentally suggest that Ror2 would serve to promote a more aggressive ccRCC. Our analysis showed a significant enrichment in high Ror2 expressing human ccRCC tumors with high nuclear grade and high clinical/tumor stage, markers for tumor progression. Additionally, we observed a significant increase of average tumor size with increased expression of Ror2. Particularly intriguing is the enrichment for Ror2-High tumors in the T4 subset. T4 tumors are defined by local invasion, and often these tumors display elements of extensive epithelial to mesenchymal transition, or even sarcomatoid histologic patterns, which was a feature highlighted in the expression profile of tumors that express the highest levels of Ror2 [4].

Recent work in melanoma has suggested a role for Ror2 in altering the cancer phenotype toward more invasive characteristics [29] in congruence with our findings presented here. However, analyses determining Ror2's potential as an independently prognostic biomarker have been limited and are not previously reported for ccRCC [11], [21]. The molecular dissection of ccRCC into two subtypes termed ccA and ccB has provided a significant improvement in determining clinical outcome (ccA-good risk, ccB-poor risk) over previous modalities centered around HIF expression [30]. Recently, this approach was further streamlined with the identification of a 34-gene biomarker geneset including Ror2 which was shown to stratify patients according to risk for disease recurrence [26]. To determine if Ror2 could serve independently in assessing clinical risk for ccRCC patients we examined the relationship of mRNA expression with outcome measures using the TCGA dataset. Our analysis of Ror2's potential as a prognostic biomarker for RCC shows that Ror2 expression is capable of independently predicting overall survival both in patients in non-metastatic and metastatic patients. Moreover, expression of Ror2 also showed significant associations with both recurrence-free survival and cancer-specific survival. Therefore, our findings highlight the role of Ror2 in promoting a more aggressive ccRCC and suggest its ability to serve as a potential independent biomarker of invasive disease in ccRCC.

## Materials and Methods

### Ethics Statement

All experiments and procedures on mice were approved and in accordance with the guidelines by the University of North Carolina at Chapel Hill Institutional Animal Care and Use Committee (IACUC) (Protocol ID 12–195.0). The University of North Carolina at Chapel Hill Institutional Animal Care and Use Committee (IACUC) approved this study.

### Cell Culture

All cells were maintained in DMEM media with 10% FBS, nonessential amino acids, L-glutamine, and penicillin/streptomycin at 37°C in 5% CO_2_. 786-0 parental cells were obtained from the American Type Culture Collection. 786-0 RC3, kindly provided by Dr. W. Kaelin (Boston, MA) were generated as previously described [31]. The stable monoclonal knockdown 786-0 shRor2.1 and shRor2.2 cells, along with the 786-0 pCDNA6 and hRor2 cells were generated were established as previously described [4]. 786-0 GFP, Ror2, and Ror2-DM cells were generated as outlined previously [17]. HEK293T is an established cell line from the ATCC. The 293T GFP, Ror2, Ror2-DM cell lines were generated as described previously [17]. Expression of all GFP tagged constructs was visually confirmed 24–48 hours after induction with doxycycline (500 ng/mL).

### Quantitative RT-PCR

Total RNA was extracted from cells using Qiagen RNeasy Mini Kit (Valencia, CA, USA). cDNA was made from 500 ng of total RNA using Random Primers (Invitrogen, Carlsbad CA) and Superscript II RT-PCR reagents (Invitrogen, Carlsbad CA) and analyzed using the ABI 7900HT Fast Real-Time PCR System with the following proprietary FAM labeled primers: Ror2, MMP2, 18S, and β-actin (Applied Biosystems, Foster City CA).

### Xenograft Analysis

50×10^4^ 786-0 or 786-0 WT8 cells were injected orthotopically with control pCDNA6 and hRor2 expressing cells into opposing kidneys in a small cohort of athymic nude (*Foxn1^nu^/Foxn1^nu^*) female mice and aged 2.5–4 months.

50×10^4^ 786-0 cells were injected subcutaneously expressing either GFP, Ror2, or Ror2 DM into a small cohort of immunodeficient (NOD.Cg-*Prkdc^scid^ Il2rg^tm1Wjl^*/SzJ) female mice and were provided doxycycline water every 2–3 days to ensure continual expression of GFP, Ror2, or Ror2-DM. Caliper measurements of the tumors were taken every 3–4 days as the mice were aged 1–1.5 months.

The resulting xenograft tumors were harvested and measured prior to being formalin-fixed and paraffin-embedded. Slides were prepared from serial sections from the prepared tissue blocks by the UNC Animal Histopathology Core. After staining with Hemotoxylin and Eosin, the prepared tissues were imaged. All studies were performed with approval of the UNC IACUC (Protocol ID 12–195.0).

### Gelatin Zymography

20 ul samples of conditioned media from samples were separated on 8% SDS-polyacrylamide gels containing 0.1% gelatin. Gels were washed in 2.5% Triton X-100 and then incubated ∼24 hours in 50 mM Tris-HCl, pH 7.5, containing 0.2 M NaCl, 5 mM CaCl_2_, and 0.02% Brij 35 at 37°C [32]. Gels were then stained using 0.5% Coomassie blue and following destaining, were imaged using the Odyssey IR imager (LI-COR Biosciences, Lincoln, NE) with densitometric analysis performed using Odyssey v3.0.21 or Image Studio on the clear bands indicating levels of the cleaved active form of MMP2 only (not the pro-MMP2 form) were analyzed.

### ELISA

Ror2 protein concentration from prepared whole cell lysates was measured using the Total Ror2 DuoSet IC ELISA (R&D Systems, Minneapolis, MN) according to the manufacturer's instructions.

### Transwell Migration/Invasion Assays

For cell migration under basal conditions, HEK293T cells were induced with doxycycline (500 ng/mL) 24 hours prior to be plated at 60×10^4^ cells, 5×10^4^ 786-0 cells for shRor2 cell lines, and 1×10^4^ 786-0 cells expressing Ror2 and Ror2-DM per well respectively in the upper chamber of an uncoated 6.5 mm 8-µm pore size transwell chamber (Corning, Corning, NY) with normal culture media in both top/bottom wells and allowed to migrate for 6 hours or 18 hours. Following migration, cells were removed from the top of the transwell by gentle swabbing. The remaining cells were fixed in 3.7% formaldehyde, washed in PBS, and stained with Crystal Violet. The percentage area covered with migrated cells was calculated across 7 random low power fields using ImageJ.

For determining cell invasion, 5×10^4^ 786-0 or 60×10^4^ HEK293T cells were plated in the upper chamber of a matrigel coated 6.5 mm 8-µm pore size transwell chamber (Corning, Corning, NY) with normal culture media in both top/bottom wells or 60 uM OA-HY (EMD Millipore, Billerica, MA) and allowed to migrate for 24 hours. Cells were fixed and imaged as previously described.

### TCGA Data Analysis

RNA sequence data was quantified using the RSEM (RNA-seq by Expectation Maximization) method [33] and normalized to the upper quartile of normal counts. Data was then log-transformed (base 2) for analysis.

### Survival Analysis

The sample data were acquired from the TCGA with appropriate permissions, and all samples were collected with appropriate consent from the source site research ethics authorities. Survival analysis was performed with the Survival library in R v 2.14, using the TCGA mRNA sequencing data [25] and associated clinical data by the clinical TCGA working group database (version April 11, 2013) and from the TCGA Biotabs database compiled on August 23, 2013. Patients with Ror2 expression above the median Ror2 expression of all samples were classified as Ror2-High and patients with Ror2 expression below the median were classified as Ror2-Low. Overall survival was defined as the time from nephrectomy to the time of death or last follow-up, the latter being censored. Cancer-specific survival was defined as the time from nephrectomy to death by ccRCC. Tumor relapse or recurrence was defined as the time from nephrectomy to the date of recurrence or metastasis. Kaplan Meier analysis were performed to assess patient probabilities for survival outcomes and log-rank scores calculated to compare Ror2-expressing groups. Log-rank p-values and statistical significance were calculated using a univariate cox regression model.

### Statistical Analysis

One-way ANOVA one-way analysis or Fisher's exact test was used to generate *p*-values in the comparison of each experimental condition with the control. A *p*-value of <0.05 was considered significant and <0.001, highly significant. All error bars shown are the calculated standard deviation (stdev) or standard error of the mean (SEM) across duplicate or triplicate experiments as indicated.

## Supporting Information

S1 Fig **Ror2 expression in various cell lines.** Total Ror2 DuoSet IC ELISA (R&D systems) shows various levels of endogenous protein expression in several established cell lines.
(TIFF)
