# Supplementary figures and images for: Expression of Ror2 Mediates Invasive Phenotypes in Renal Cell Carcinoma
Source: PLoS One. 2014 Dec 26;9(12):e116101. doi: 10.1371/journal.pone.0116101 (PMC4277431; doi:10.1371/journal.pone.0116101)

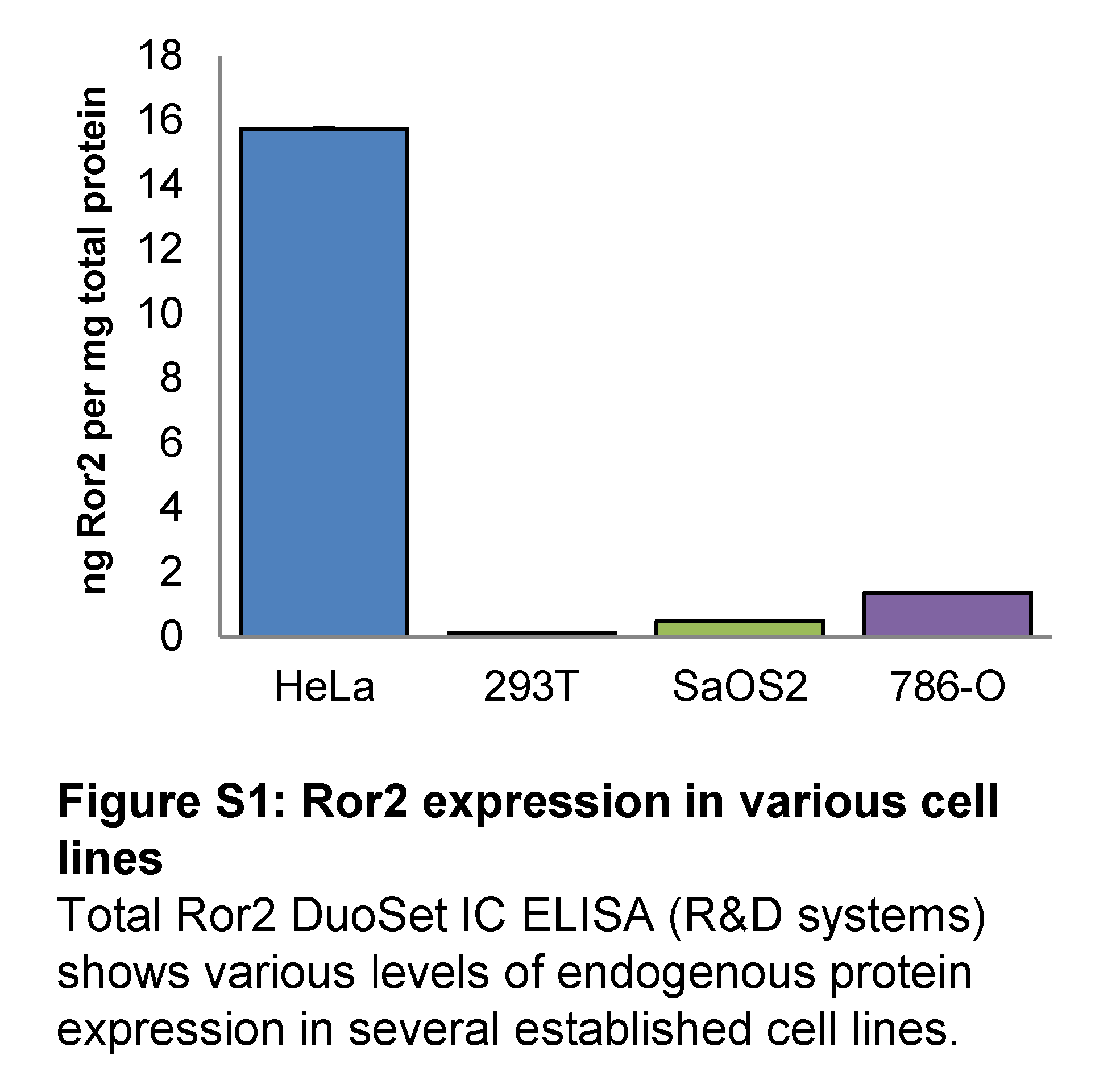

Supplement: S1 Fig — Ror2 expression in various cell lines. Total Ror2 DuoSet IC ELISA (R&D systems) shows various levels of endogenous protein expression in several established cell lines. (TIFF) [file pone.0116101.s001.tiff]
